# Investigating the Role of Known Arabidopsis Iron Genes in a Stress Resilient Soybean Line

**DOI:** 10.3390/ijms252111480

**Published:** 2024-10-25

**Authors:** Jamie A. O’Rourke, Michelle A. Graham

**Affiliations:** Agricultural Research Service, Corn Insects, and Crop Genetics Research Unit, United States Department of Agriculture, Ames, IA 50010, USA; michelle.graham@usda.gov

**Keywords:** soybean, iron deficiency, RNAseq, Fiskeby III

## Abstract

Genes involved in iron deficiency responses have been well characterized in *Arabidopsis thaliana*, but their roles in crop species have not been well explored. Reliance on model species may fail to identify novel iron stress mechanisms present within crop species, likely selected by hundreds of years of selection. Fiskeby III (PI 438471) is a soybean line from Sweden that demonstrates high levels of resilience to numerous stresses. Earlier Fiskeby III studies have identified a suite of genes responding to iron deficiency stress in Fiskeby III that are also associated with Arabidopsis iron deficiency responses. We were interested in determining how canonical iron genes function in Fiskeby III under normal and iron stress conditions. To investigate this, we used virus-induced gene silencing to knock down gene expression of three iron deficiency response genes (FER-like iron deficiency induced transcription factor (FIT), elongated hypocotyl 5 (HY5) and popeye (PYE)) in Fiskeby III. Analyses of RNAseq data generated from silenced plants in iron-sufficient and -deficient conditions found silencing FIT and HY5 altered general stress responses but did not impact iron deficiency tolerance, confirming Fiskeby III utilizes novel mechanisms to tolerate iron deficiency stress.

## 1. Introduction

Iron (Fe) is the fourth most abundant mineral in the earth’s crust. However, the majority of iron is present as Fe^3+^, which is not biologically available to plants. Plants can only directly utilize iron in the Fe^2+^ form, which is much less prevalent in the soil than Fe^3+^. Many environmental factors including weather conditions and soil properties can shift the balance of Fe^3+^ to Fe^2+^. Plants have evolved two strategies to handle low iron conditions, strategy I and strategy II [1]. Strategy I is a reduction-based approach. Plants excrete protons via Arabidopsis H^+^ ATPase2 (AHA2) to acidify the rhizosphere. Next, a root membrane bound reductase ferric reduction oxidase 2 (FRO2) reduces Fe^3+^ to Fe^2+^. This usable iron is then transported into the root of the plant by iron-regulated transporter 1 (IRT1). Strategy II is chelation-based. Strategy II plants release phytosiderophores, members of the mugineic acid family, from the roots to solubilize and bind Fe^3+^ and the entire complex is then transported into the roots. It was originally proposed that monocot species use strategy II, while dicot species use strategy I. However, recent findings have demonstrated that dicots can induce some aspects of the strategy II response [2,3]. Current evidence suggests soybean utilizes the strategy I response.

The research in our labs has focused on the soybean line Clark (PI548533), an iron efficient line. In our studies we have found Clark has a unique response to iron deficiency (−Fe) stress, inducing genes associated with stress, defense, and iron uptake and transport genes while repressing genes associated with DNA replication, methylation, and photosynthesis [4,5,6,7,8,9,10]. The iron efficiency of Clark is conferred by a quantitative trait loci (QTL) on GmChr03 [11,12,13], while the Fiskeby III efficiency is conferred by a QTL on GmChr05 [14]. Given the high resilience of Fiskeby III to multiple stresses and the different sources of the iron stress tolerance, we were interested in determining how Fiskeby III iron stress tolerance would be impacted by silencing major genes involved in the iron stress response.

Most knowledge of strategy I responses comes from studies in *Arabidopsis thaliana*. However, the findings from Arabidopsis have not directly translated to soybean. In this study, we utilized virus-induced gene silencing (VIGS) to down-regulate three of the key genes in the Arabidopsis −Fe response pathway: FER-like iron deficiency-induced transcription factor (FIT), elongated hypocotyl 5 (HY5), and popeye (PYE) in the Fiskeby III soybean line. These genes were chosen because they are either differentially expressed in Fiskeby III due to iron deficiency stress (PYE, [15]), or are believed to regulate (based on Arabidopsis) DEGs identified in earlier Fiskeby III studies (FIT and HY5, [15,16]). Understanding if and how these genes function in soybean, particularly a line that is so stress resilient, will help us understand how soybean modifies gene expression profiles to address nutrient deficiencies.

Basic-helix-loop-helix (bHLH) transcription factors (TFs) regulate many facets of strategy I iron deficiency response. One of the major bHLH TFs associated with −Fe is FIT (*At2g28160*) [17,18]. In Arabidopsis, FIT is a key regulator of Fe acquisition, serving as a central hub to integrate signals from developmental and environmental pathways to maintain Fe homeostasis [19]. In *fit* knockout mutants, plants are iron deficient, even when grown on iron replete media [20]. FIT regulates the expression of the main strategy I genes including *AHA2*, *FRO2*, and *IRT1* [21]. Under −Fe conditions, a suite of bHLH TFs including bHLH038, bHLH039, bHLH100, and bHLH101 directly interact with FIT to activate FIT [18,22]. In earlier Fiskeby III studies, *bHLH038* was differentially expressed in response to iron [16]. As these data were collected after 16 days in hydroponics, we hypothesize that FIT may be associated with the regulation of *bHLH038* in Fiskeby III.

A second important bHLH TF is POPEYE (PYE, *At3g947640*). PYE regulates Arabidopsis growth and development during −Fe stress [23]. Originally, PYE was thought to regulate Fe homeostasis independently of FIT. However, it has since been found that PYE directly interacts with bHLH TFs, including bHLH038/039/100/101/104/105/115. It is well known that bHLH038 and bHLH039 interact with FIT [18]. As PYE represses bHLH38/39 transcription, PYE is not acting completely independently of FIT [24]. In Arabidopsis, PYE expression is induced by −Fe and is required to inhibit the excessive induction of Fe homeostasis-associated genes [23]. PYE also serves to protect plants from light damage, including reactive oxygen species and the destruction of photosynthetic machinery. Specifically, under −Fe, the grana in Arabidopsis thylakoids de-stack, but in PYE mutants the grana remain stacked [25]. Previous experiments silencing a candidate gene associated with the GmChr05 QTL found PYE was up-regulated in leaves compared to EV in both +Fe and −Fe conditions [15]. 

Elongated Hypocotyl 5 (HY5, *At5g11260*) is a bZIP TF that was originally identified as an important component of light responses and the regulation of seedling development [26]. Further studies revealed HY5 plays multiple roles in plant development, signaling, and stress responses [27]. HY5 is unique in that it is a shoot-to-root mobile signal that co-ordinates plant carbon and nitrogen uptake [28]. HY5 is also involved in multiple hormone signaling pathways including gibberellic acid, brassinosteroids, ethylene, auxin, and abscisic acid, all of which have been implicated in −Fe responses in multiple species [27,29]. In Arabidopsis, HY5 is known to be involved in regulating −Fe responses, regulating the expression of multiple genes involved in iron uptake and homeostasis [30], and was recently shown to directly interact with both FIT and PYE [31,32] to regulate iron homeostasis. This is reaffirmed in Arabidopsis HY5 knockout plants, which are stunted with chlorotic leaves under −Fe conditions. In previous work by our lab, the HY5 transcription factor binding site was over-represented amongst the promoters of differentially expressed genes, indicating a role for HY5 in soybean −Fe responses as well [5,15,16]. Together, these three genes control a large swath of genes associated with the strategy I −Fe responses in Arabidopsis. However, an improved understanding of these genes in soybean, an agronomically important crop, is important to developing more resilient plants. Fiskeby III is the most abiotic stress-resilient soybean line we have identified. An improved understanding of how these genes function in Fiskeby III could help us translate the stress tolerance of Fiskeby III into elite breeding lines.

## 2. Results

Viral constructs were tested in both Williams 82 and Fiskeby III soil-grown plants. Williams 82 was utilized since it carries the historical IDC QTL from Gm03. Comparing the VIGS constructs in soil-grown Williams 82 plants to EV-infected plants found significantly higher SPAD levels for VIGS_FIT plants and significantly lower SPAD levels for VIGS_HY5 plants. VIGS_HY5 plants also showed significantly reduced height and weight (Figure S1). There were no statistically significant changes in VIGS_PYE plants for any of the phenotypic measurements used. In soil-grown Fiskeby III plants, there were no statistical differences among any of the phenotypic measurements for any of the silenced plants Because these genes are important in iron-stress responses, we were most interested in whether these genes showed a phenotype in iron-stressed plants, which we generated using hydroponics. Like the soil-grown plants, there were no statistically significant changes in phenotypic measurements for any of the silenced plants grown in +Fe and −Fe hydroponics. Given the high resilience of Fiskeby III to multiple abiotic stresses, including iron deficiency, the lack of phenotypic changes was not surprising. If the candidate genes identified from Arabidopsis play a role in the Fiskeby III iron-stress response, we expect to see transcriptional changes in response to silencing, and a sub-set of these changes should be unique to each of the constructs, despite a lack of phenotypic change. Based on our previous research, we know iron stress-tolerant soybean (genotype, Clark) exhibits strong gene expression changes within one hour of iron stress exposure [6,7]. Therefore, we used RNA-seq to characterize Fiskeby III responses to HY5, FIT, and PYE silencing one hour after iron-stress exposure. These transcriptional changes should provide insight into the iron-stress tolerance of Fiskeby III.

### 2.1. Analyses of RNAseq Data

#### 2.1.1. Silencing Effect

**Figure 1:**
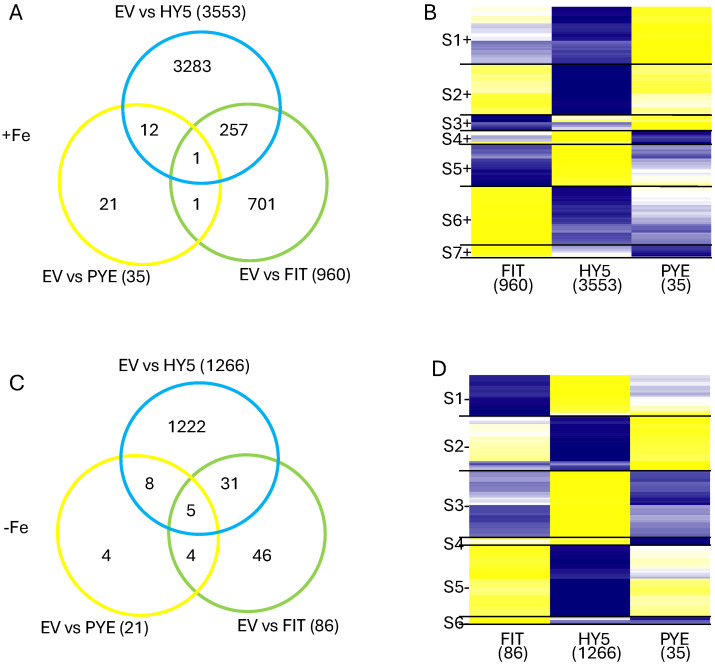
Intersection of DEGs from the silencing effect analyses. Venn diagrams and corresponding heatmaps represent EV vs. VIGs silenced plants in either +Fe conditions (**A**,**B**) or –Fe conditions (**C**,**D**). DEGs were identified as those with an FDR < 0.05. The Venn diagrams (**A**,**C**) identify genes unique to an analysis or genes differentially expressed in multiple analyses. The cluster analyses to generate heatmaps (**B**,**D**) were performed in R to group genes by similar expression patterns. Genes in yellow are induced while those in blue are repressed. These groupings are delineated by the horizontal black lines, and each cluster is assigned a unique designation to the left of the heatmap; S1+–7+ represent silencing effect analyses in +Fe conditions, S1–6—represent genotype analyses in –Fe conditions. The total number of DEGs identified by each analysis is provided outside the circles of interest (**A**,**C**) in the Venn diagrams and below the column of interest in the heatmaps (**B**,**D**).

The design of the RNAseq experiment allowed us to make two different types of comparison, silencing effect and iron treatment effect (described below). The silencing effect contrasted VIGS_HY5, VIGS_FIT, or VIGS_PYE to EV, examining +Fe and −Fe separately. The silencing effect would identify genes differentially expressed, directly or indirectly, in response to the silencing of HY5, FIT, or PYE in the different conditions (Tables S1 and S2). The total number of identified DEGs for both comparisons are listed in Table 1. The distribution of the DEGs identified is also visualized by Venn diagrams in Figure 1A,C.

Contrary to our expectations, there were fewer DEGs identified in EV vs. VIGS plants in −Fe conditions than in +Fe conditions. VIGS_HY5 vs. EV in both +Fe and −Fe conditions identified the most DEGs in the silencing effect analyses. Further analysis of the RNA-seq data from the silenced plants should identify novel strategies utilized by Fiskeby III to counter iron stress. We used the GO tool (version 4) on soybase.org (accessed on 3 March 2023) to identify over-represented (corrected *p* < 0.05) GO terms for all DEGs within a VIGS construct of interest (Table S3). Within a DEG list, DEGs were divided into those induced or repressed by silencing. The 637 DEGs induced when comparing VIGS_FIT to EV plants in +Fe conditions were largely associated with photosynthesis (15 GO terms) including GO terms photosynthesis, thylakoid membrane organization, light reaction, chlorophyll biosynthesis, carotenoid biosynthesis, and response to light. This likely reflects the importance of iron ions in the electron transport chain. Other induced GO terms included those associated with fatty acid biosynthesis, response to hormones (auxin and jasmonic acid stimulus), divalent metal ion transport, defense response-incompatible reaction, cellular cation homeostasis, ammonia assimilation cycle, and iron-sulfur cluster assembly. The 324 DEGs repressed by FIT silencing in +Fe conditions included 15 GO terms associated with DNA replication, DNA methylation, cell cycle regulation, and gene silencing. Given the few DEGs identified for the same comparison under −Fe conditions, it is not surprising there are no statistically significant GO terms identified. However, by comparing DEGs directly, we identified 17 DEGs common to FIT-silenced plants in −Fe and +Fe, many with Arabidopsis homologs with functions related to stress tolerance, immunity, and/or abscisic acid signaling. AtPYL4 (Glyma.01g124700, Glyma.06g039000, and Glyma.07g155500) is involved in ABA signaling and drought-resistance [33]. AtATL6 (Glyma.09g140700) and AtNDL1 (Glyma.20g223800) regulate responses to salt stress [34,35]. AtLYK3 (Glyma.08g200800) regulates plant immunity [36] and abscisic acid signaling.

For DEGs responding to silencing in VIGS_HY5 vs. EV under +Fe conditions, we identified 2612 repressed DEGs corresponding to 70 statistically significant GO terms. A large number of GO terms (20) were associated with DNA replication, DNA methylation, cell cycle regulation, and gene silencing. We also identified significant GO terms associated with fatty acid, sterol, flavonoid, flavanol, phenylpropanoid, anthocyanin, polysaccharide, jasmonic acid and lipid biosynthesis, lipid oxidation, and response to UV, sucrose stimulus, wounding, cadmium, Karrikin, and cylopentenone. The 941 DEGs induced by HY5 silencing in +Fe were associated with two significant GO terms: production of siRNA involved in RNA interference and autophagy.

Under −Fe conditions, we identified 684 DEGs repressed by HY5 silencing corresponding to 28 statistically significant GO terms. Of these, 14 GO terms associated with DNA replication/methylation/cell cycle and photosynthesis were also significant among HY5 silencing repressed genes in +Fe conditions. The 582 DEGs induced in −Fe conditions were associated with silencing (post-transcriptional and virus-induced gene silencing). It is worth noting that 704 DEGs were shared by HY5 silencing in −Fe and +Fe conditions. Unique to the −Fe analyses is the GO term ‘divalent metal transport’, which is associated with 21 DEGs. Examining the annotation of these genes indicates these genes are associated with photosynthesis, likely due to the high iron requirement of the electron transport chain and chlorophyll formation.

No significant GO terms were identified for any VIGS_PYE analyses, likely due to the small number of DEGs identified. This suggests silencing PYE had little impact on Fiskeby III gene expression. This could mean that PYE does not have a role in iron stress responses in leaves or, alternatively, due to the polyploid nature of the soybean genome, gene redundancy could mask the impact of silencing.

To visualize the expression patterns of the genotype analyses, we used RStudio (version 4.3.2) to conduct hierarchical clustering and build heatmaps (Figure 1B,D). For each heatmap, we created a nonredundant list of all DEGs identified when comparing VIGS_FIT, VIGS_HY5, and VIGS_PYE to EV in +Fe conditions or −Fe conditions. In +Fe conditions, clusters S2+ (882 DEGS), S4+ (247 DEGs), and S5+ (705 DEGs) were made up of DEGs significant only in VIGS_HY5 plants (>94.7%). Cluster S2+ was associated with a broad range of GO terms including the biosynthesis of fatty acid, sterol, brassinosteroid, flavonoid, coumarin, anthocyanin, and polysaccharide. Cluster S4+ had no significant GO terms, while cluster S5+ was associated with gene silencing (Table S5). Cluster S1+ (973) was also largely composed of VIGS_HY5 DEGs, however, 106 were also significant in VIGS_FIT, with expression patterns conserved across constructs. This cluster was associated with GO terms related to DNA replication/methylation and the cell cycle. Clusters S3+, S6+, and S7+ were composed with DEGs from both VIGS_HY5 and VIGS_FIT. In cluster S3+, 70 DEGs were common to VIGS_HY5 and VIGS_FIT plants, and expressed similarly. The 1004 DEGs in cluster S6+ was composed of 467 DEGs from VIGS_FIT, and 581 DEGs from VIGS_HY5. Similarly, of the 213 DEGs in cluster S7+, 128 came from VIGS_FIT and 93 came from VIGS_HY5. In Fe- conditions, more than 90% of DEGs within a cluster were only significant in VIGS_HY5, highlighting what little impact the VIGS_FIT and VIGS_PYE constructs had in −Fe conditions.

#### 2.1.2. Treatment Effect

**Figure 2:**
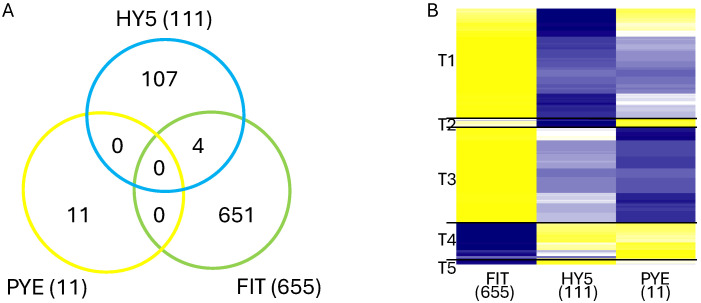
Intersection of DEGs from the treatment effect analyses. Venn diagrams and corresponding heatmaps represent DEGs identified from comparing VIG-silenced plants grown in −Fe vs. +Fe. DEGs were identified as those with an FDR < 0.05. Venn diagram (**A**) identifies genes unique to a single candidate gene analysis or genes differentially expressed by silencing multiple candidate genes. The cluster analyses to generate the heatmap (**B**) were performed in R to group genes by similar expression patterns. Genes in yellow are induced, while those in blue are repressed. These groupings are delineated by the horizontal black lines, and each cluster is assigned a unique designation to the left of the heatmap (T1–5). The total number of DEGs identified by each analysis is provided outside the circle of interest (**A**) in the Venn diagram, and below the column of interest in the heatmap (**B**).

The treatment effect contrasted −Fe vs. +Fe for EV, VIGS_FIT, VIGS_HY5, and VIGS_PYE plants separately. This would identify genes differentially expressed in response to iron availability in specific VIGS backgrounds. If silencing had no effect on iron responsiveness, we would expect the VIGS_FIT, VIGS_HY5, and VIGS_PYE constructs would have the same DEGs as the EV construct. However, if silencing impacted iron stress responsiveness, different DEGs could be identified when comparing EV DEGs to the other VIGS constructs. Not surprisingly, given the high stress tolerance of Fiskeby III, no DEGs were identified in EV −Fe vs. +Fe (Table 1). Therefore, the 11, 111, and 655 DEGs identified in PYE, HY5, and FIT plants, respectively, became iron-responsive as a result of silencing. This suggests all three genes regulate, directly or indirectly, iron-responsive genes in Fiskeby III. Overall, the treatment effect analyses identified fewer genes than silencing effect analyses (Table 1 and Table S6 and Figure 2). Of the 655 DEGs responding to iron treatment in FIT-silenced plants, 379 were unique to the iron treatment response, confirming FIT-silencing altered the iron responsiveness of these genes. Comparing DEGs responding to HY5-silencing relative to EV in +Fe and −Fe conditions to DEGs responding to iron treatment, we identified only 13 unique DEGs. This suggests HY5 function is important in both +Fe and −Fe conditions. As in the silencing effect analyses, the treatment analyses for VIGS_PYE failed to identify any over-represented GO terms.

Mirroring our analyses of the silencing effect, each of the three treatment gene lists was divided by induced or repressed. The induced and repressed gene lists were then used with the GO tool on SoyBase to identify over-represented (Corrected *p* < 0.05) GO terms. The 538 induced genes from the VIGS_FIT analyses were mainly associated with photosynthesis (15 GO terms) including photosynthesis, response to light, and chloroplast organization. Again, reflecting the importance of iron in photosynthetic processes. There were no GO terms associated with the 117 repressed genes in VIGS_FIT. The VIGS_HY5 analysis is unique in that, of the 111 DEGs identified, 109 were repressed. Five of the eight GO terms identified from the 109 repressed genes are associated with fatty acids and wax, including lipid biosynthesis, cuticle development, fatty acid metabolism, cutin biosynthesis, and lipid metabolism. Finally, the PYE analyses identified 11 DEGs, all induced. Five of the 11 genes are associated with nucleosome assembly processes.

As with the DEGs identified from the silencing effect analyses, we used a non-redundant list of treatment DEGs to build a heatmap using RStudio (version 4.3.2) (Figure 2B). This heatmap showed that, like the silencing effect heatmaps, the expression pattern of genes in FIT-silenced plants are dramatically different from those in HY5. Cluster T1 is composed of genes from both FIT (249 DEGs) and HY5 (85 DEGs) with four overlapping genes, but expressed in the opposite direction. These 330 DEGs are associated with 31 GO terms, 13 of which are associated with photosynthetic processes including chlorophyll biosynthesis, response to light, and thylakoid organization. Genes in cluster T2 are unique to HY5, while genes in clusters T3 and T4 are unique to FIT. There are no statistically significant GO terms associated with T2, likely due to the few genes in the cluster. Cluster T3 (289 DEGs) is, again, associated with photosynthesis (six GO terms) but also DNA replication/methylation (four GO terms). The only statistically significant GO term associated with T4 is nucleosome assembly. Finally, T5 (20 DEGs, 18 from FIT and 2 from HY5) are associated with reciprocal DNA recombination. Notably absent were any GO categories related to iron uptake, transport, or metabolism.

#### 2.1.3. Known Iron Genes

Given the lack of GO categories involved with iron uptake/transport, we examined the annotations associated with the Arabidopsis homologs of each of the DEGs in all nine (silencing effect and treatment) comparisons. In the silencing effect −Fe analyses (Table S2), only two classic iron genes were identified. The first was *Glyma.15G274600*, a homolog of *FRD3* (*At3G08040*), which is a plasma membrane transporter that is important for iron distribution and homeostasis [37]. In our experiment, *FRD3* expression is induced in VIGS_HY5 plants compared to the EV plants under both −Fe and +Fe conditions. The second, a homolog of *HY5* (*Glyma.04G225300*, which shares <80% of the sequence identity with the VIGS construct sequence), was repressed. The lack of additional iron-specific genes, either induced or repressed, under −Fe conditions may indicate that the timing of the sampling was such that we failed to capture the expression of these genes in leaves or possibly that the resilience of Fiskeby III means 60 min of −Fe stress is not enough to induce the canonical genes.

In contrast to the two iron-specific DEGs identified under −Fe conditions, in +Fe conditions, silencing effect analyses identified 28 homologs of genes known to be involved in the iron stress-response (Table S1). All 28 DEGs were identified in the VIGS_HY5 vs. EV +Fe comparison. These genes include homologs of *BRUTUS (BTS*, *Glyma.07G093700* and *Glyma.09G182600*), *FIT* (*Glyma.12G178500*), *bHLH038* (*Glyma.03G130400* and *Glyma.03G130600*), *bHLH121* (*Glyma.08G148500*), *ORG1* (*Glyma.04G236600*), *OPT3* (*Glyma.17G006100*), *IREG1* (*Glyma.01G128300*), *HY5* (*Glyma.08G302500*), *FRD3* (*Glyma.15G274600*), and *NRAMP3* (*Glyma.05G101700* and *Glyma.17G165200*). The homologs of *bHLH121*, *IREG1*, *HY5*, and *FRD3* are all induced in HY5-silenced plants compared to EV. Conversely, the homologs of *BTS*, *FIT*, *bHLH038*, *ORG1*, *OPT3*, and *NRAMP3* are all repressed in VIGS_HY5 plants. Three additional iron-associated genes were identified, one, a homolog of *PYE* (*Glyma.17G098900*), is induced in VIGS_PYE vs. EV +Fe analyses, possibly an artifact of the silencing process since this gene shares >80% nucleotide identity to the silenced PYE gene. The two remaining genes of interest are heavy metal transporters (*Glyma.19G012900* and *Glyma.19G209500*), induced and repressed, respectively, in VIGS_FIT vs. EV +Fe comparisons.

The treatment analyses (Table S6) only identified four iron-specific genes, all four of which are induced in the FIT analysis. Two of the genes are generic iron genes: a vacuolar iron transporter (VIT) responsible for transporting excess iron in and out of the vacuole (*Glyma.04G225300*), and a cation transporter, which could transport a wide range of cations beyond iron (*Glyma.15G262300*). Glyma.10G146600 encodes IREG3, an iron transporter important in maintaining cellular ion homeostasis [38]. Finally, Glyma.09G018600 encodes a homolog of Arabidopsis ferric reduction oxidase 6 (FRO6). In tobacco, overexpressing FRO6 increases iron deficiency tolerance [39].

In addition to looking for iron genes identified from Arabidopsis, we compared the DEGs identified in this study to the DEGs identified from eight previous studies by our group using multiple genotypes and multiple timepoints (30 min, 60 min, 120 min, 1 day, 2 days, 7 days, 10 days, 16 days) [5,7,8,9,10,15,16,40]. Comparing the DEGs in this study to these publications identified 54 DEGs from the silencing +Fe analyses, 27 DEGs from the silencing −Fe analyses, and 20 DEGs from the treatment analyses that were also differentially expressed in an earlier study (Table S7). Overwhelmingly (>80%), the DEGs were identified from a previous study using Fiskeby III where plants were subjected to 16 days of −Fe stress. The lack of overlap with other studies confirms the Fiskeby III utilizes novel stress response pathways.

Studies have shown that iron-deficient plants produce high levels of reactive oxygen species (ROS), which may serve as an iron-deficient signal or serve as part of ROS-scavenging enzymes [41,42,43,44,45]. No GO terms associated with ROS or oxidation stress were over-represented in any of our analyses. However, querying individual lists for genes associated with ROS oxidative stress (catalases, superoxide dismutases, ascorbate peroxidases, glutathione, and peroxiredoxins) identified 79 DEGs in silencing +Fe, 30 DEGs in silencing −Fe, and 11 DEGs in the treatment analyses (Table S8). In both silencing analyses, the majority of the oxidative stress genes are associated with glutathione. Glutathione, with ascorbic acid, acts to maintain redox balance through scavenging and removal of hydrogen peroxide. Under −Fe conditions, increased glutathione and ascorbic acid protect against −Fe by preserving chlorophyll [46] and allow plants to grow as if in +Fe conditions. In our data, most glutathione-associated genes were identified in the VIGS_HY5 plants under +Fe and −Fe conditions, and are repressed in the VIGS_HY5 plant compared to VIGS_EV. The repression of these genes in silenced plants under both +Fe and −Fe conditions indicate they are not unique to iron stress and may play a role in general stress responses.

#### 2.1.4. Potential Regulation of Fiskeby III Responses

To understand the regulation of the DEGs identified in this study, we used CLOVER [47] to identify any over-represented transcription factor binding sites (TFBS) in the 1000 bp promoters of the DEGs (Table 2). To maximize both computational resources and biological insights, we analyzed induced and repressed genes of each list separately (Table S4). For VIGS_FIT vs. EV in +Fe we identified 64 TFBS motifs over-represented in the promoters of induced genes and 27 TFBS motifs in the promoters of repressed genes. The motifs are associated with 617 induced genes and 319 repressed genes, which is nearly all the DEGs for the FIT gene silencing analyses. The induced genes are largely associated with photosynthetic processes, while the repressed genes are associated with DNA replication and methylation processes. For VIGS_FIT vs. EV in −Fe we identified three over-represented TFBS associated with induced genes and three different TFBS associated with the repressed genes. The motifs are associated with 31 repressed genes and 36 induced genes. The VIGS_FIT treatment comparison identified 44 TFBS over-represented in the promoters of the 518 induced genes and two motifs over-represented in the promoters of 117 genes. Comparing the silencing effect to the treatment effect found 40 of the 44 TFBS were conserved between treatment and VIGS_FIT +Fe comparisons among the induced genes, representing 246 of the 262 genes conserved between the two comparisons.

For VIGS_HY5 vs. EV in +Fe, we identified 144 TFBS motifs identified in promoters of the repressed genes (2612), 35 of which were MYB TFBS (Table 2 and Table S4). The 144 statistically significant TFBS identified correspond to 2210 DEGs, nearly all of the 2612 DEGs. The 35 MYB TFBS were identified in the promoter regions of 1293 of the DEGs. These 1293 DEGs are associated with DNA replication/methylation, growth, and defense. These results suggest HY5 is involved in regulating MYB TFs associated with repressing the expression of these genes. The CLOVER results of VIGS_HY5 vs. EV in −Fe conditions identified 15 TFBS in down-regulated genes and 58 TFBS in up-regulated genes. Two TFBS were conserved between up- and down-regulated genes: an ABA-insensitive 5/Dc3 promoter binding factor 3 (DPBF3) binding site (MA1338.2) and a HY5 binding site (MA0551.1). DPBF3 TF is associated with abscisic acid (ABA), including regulating interactions between ABA and sugar signaling and regulating response to ABA and drought stress [48,49]. The HY5 TFBS was identified in the promoter of 50 down-regulated and 43 up-regulated genes. Not surprisingly, given the role HY5 plays in light reactions [29,31,50], the promoters with the HY5 TFBS are associated with genes involved in photosynthesis. Identifying the HY5 TFBS in the promoter of DEGs from VIGS_HY5 plants confirms that the DEGs are associated with HY5.

The treatment (−Fe vs. +Fe) analyses of VIGS_HY5 plants identified 111 DEGs (Table 1). CLOVER analyses identified 17 over-represented TFBS (Table 2), including eight MYB TFBS in the 109 promoters of repressed genes. The MYB TFBS are identified in 101 of the 109 down-regulated genes. The majority of these genes (87) were also repressed in VIGS plants compared to EV. These genes are associated with lipid and flavonoid biosynthesis. The only differentially expressed MYB in the treatment comparison is MYB94, which is associated with inducing cuticular wax. It has been well documented that HY5 directly regulates MYB TFs in response to light. However, this is the first evidence that HY5 is regulating MYBs in response to iron deficiency.

## 3. Discussion

The iron uptake system has been well characterized in model species. However, translating the findings from model species to crop species has proven inefficient. This knowledge gap means iron deficiency is still a major issue for soybean growers, as increasing iron deficiency correlates with increasing yield loss [51,52]. The soybean genotype Fiskeby III is unique in its tolerance to multiple abiotic stresses. Previous studies have identified multiple iron tolerance QTL, including the canonical soybean iron QTL on GmChr03 and a novel QTL on GmChr05. While improved iron tolerance has been mapped to these regions, how plants achieve improved tolerance is still not understood. In comparing DEGs from this study to previous publications that focused on the GmChr03 QTL, there was little overlap of specific DEGs, though the same GO categories were identified. We believe the GmChr03 QTL in Fiskeby III serves as a secondary iron homeostasis system, and that Fiskeby III overwhelmingly utilizes the GmChr05 mechanisms. While breeders can integrate the QTLs, more targeted selection is desirable. Therefore, identifying specific genes of interest will help breeders select lines with improved tolerance and associated yield. Given the increased tolerance of Fiskeby III to multiple abiotic stresses, we were interested in seeing whether the canonical genes identified from Arabidopsis for iron deficiency tolerance would impact the iron tolerance of Fiskeby III. Accordingly, we used VIGS to silence the homologs of three canonical genes associated with iron deficiency stress in Arabidopsis (PYE, HY5, and FIT) in Fiskeby III grown in soil and in iron-sufficient and -deficient hydroponics. In addition, we conducted RNAseq analyses to determine how silencing these genes impacted gene expression in Fiskeby III.

Silencing FIT in Williams 82 soil-grown plants resulted in significant differences in SPAD values. In previous soybean research, SPAD values have been successfully used to map quantitative trait loci for iron deficiency chlorosis. In this study, VIGS_FIT plants had significantly higher SPAD levels than the EV control plants, suggesting silencing FIT improved plant health. In Arabidopsis, *fit*1 seedlings were chlorotic and died two to three weeks after germination [21] Silencing FIT in Fiskeby III had no significant phenotype in soil or hydroponic conditions. Interestingly, silencing FIT identified many of the same genes that have been previously shown to respond to iron stress in the IDC-tolerant line Clark. The repression of genes involved in DNA replication/methylation and photosynthesis and induction of genes associated with iron uptake and homeostasis, defense, and stress are hallmarks of the Clark iron stress response. In this study, the silencing of FIT in iron-sufficient conditions repressed genes associated with DNA replication/methylation, and induced genes associated with photosynthesis and defense. Interestingly, we failed to identify genes responding to FIT silencing that were associated with iron uptake and homeostasis. To confirm this response, we used the best Arabidopsis homologs of DEGs responding to FIT silencing to cross-reference the 34 robustly FIT-regulated genes identified by Mai et al. [46]. None of the FIT-regulated genes identified by Mai et al. [46] was identified in our analyses. This suggests that, in soybean, FIT is a negative regulator of iron stress responses. This is supported by the 655 DEGs that became iron-responsive in FIT-silenced plants. In particular, FIT appears to regulate the iron responsiveness of photosynthetic genes.

Silencing HY5 in Williams 82 plants grown in soil resulted in significantly lower SPAD levels, reduced plant height, and reduced plant weight. This is consistent with findings by Mankotia et al. [30] who reported HY5 mutants had reduced chlorophyll content and growth under iron stress conditions. This would suggest that HY5 is a positive regulator of iron stress responses in Williams 82. However, the silencing of HY5 in Fiskeby III had no significant phenotype in soil or in iron-sufficient or -deficient hydroponics. As above, RNAseq analysis revealed HY5 silencing resulted in the repression of DEGS associated with DNA replication/methylation and photosynthesis in iron-sufficient and -deficient conditions. This mirrors expression patterns observed by Atwood et al. [4], who used the silencing of the DNA replication gene *GmRPA3c* to relieve iron stress symptoms in the iron stress susceptible line IsoClark. This suggests that HY5 is a negative regulator of iron stress responses in Fiskeby III. In particular, 105 DEGS associated with the biosynthesis of lipids, long-chain fatty acids, cutin, and flavonoids were repressed by iron stress due to HY5 silencing.

In contrast to silencing HY5 and FIT, silencing PYE had no phenotype in either Williams 82 or Fiskeby III. In Arabidopsis, the leaves of *pye-1* plants became chlorotic when seeds were germinated on iron-deficient media [23]. When transferred from sufficient to deficient conditions, *pye-1* plants had reduced chlorophyll content relative to wild type plants. In Arabidopsis, PYE represses the transcription of *bHLH38*, *bHLH39*, *bHLH100*, and *bHLH101* which interact with FIT to induce iron stress-responses [24]. While the redundant functions of *bHLH38*, *bHLH39*, *bHLH100*, and *bHLH101* have long been recognized, refs. [40,53] recently demonstrated the four genes lie in three syntenic blocks, helping to explain their co-regulation by PYE. Interestingly, only two homeologous genes with significant sequence homology and synteny to *bHLH38*, *bHLH39*, *bHLH100*, and *bHLH101* are present in the soybean genome. Silencing both homologs (syntenic to bHLH28) via VIGS resulted in no phenotype in iron-sufficient conditions. Combined with our results, this suggests this complex of genes does not function in soybean as predicted by reviewing the Arabidopsis literature.

Fiskeby III’s ability to tolerate iron stress is confirmed by the fact that no DEGs responding to iron stress were identified from EV plants. While silencing FIT, HY5, and PYE had no phenotypic impact on Fiskeby III, they did help identify genes and pathways that could be contributing to Fiskeby III’s iron stress-tolerance. In our study, the treatment analyses of HY5 identified 109 repressed genes largely associated with fatty acid and wax biosynthesis, including cuticle development and cutin biosynthesis. Whaley et al. [54] hypothesized that abiotic stress tolerance of Fiskeby III was associated with the altered leaf physiology, providing passive resistance to ozone stress exposure. They found increased levels of wax and cutin biosynthetic genes in Mandarin (Ottawa) leaves compared to Fiskeby III, hypothesizing that increased expression in Mandarin (Ottawa) was an effort by the plant to increase the physical barrier to ozone penetration of the leaves. Our findings suggest these pathways are turned on very early in response to iron stress in Fiskeby III. In pear (*Pyrus communis*) and peach (*Prunus persica*), −Fe conditions reduce cuticular wax in leaves, thus altering water relations, and pest and disease resistance [55].

Given the large number of DEGs identified in many of our comparisons, we compared the gene lists to see if the response of Fiskeby III to the silencing of each of the three genes was conserved (Figure 1 and Figure 2). In the silencing effect comparisons (VIGS vs. EV), only a small number of the genes are differentially expressed in multiple gene lists (Figure 1). Under +Fe conditions, only a single gene is differentially expressed in all three comparisons. This gene (*Glyma.09G172500*) corresponds to Arabidopsis map kinase kinase 9 (*At1g73500*), which positively regulates salt tolerance in Arabidopsis [56,57]. Under −Fe conditions, there are five genes differentially expressed in all three comparisons, and four of the Arabidopsis homologs are closely involved in defense/stress responses. These four genes include homologs of KTI1 (*Glyma.08G235400*, *At1g73260*), which modulates programmed cell death in plant/pathogen interactions; RIC1 (*Glyma.10G251400*, *At2g33460*), which interacts with ROP2 to fine-tune microtubule dynamics in response to salt stress; a serine proteinase inhibitor (*Glyma.20G205800*, *At2g38870*), which is involved in plant defense by encoding pathogenesis related proteins; and HKL1 (*Glyma.11G15800*, *At1G50460*), which is a negative regulator of plant growth and a positive regulator of immune responses [58,59,60,61,62]. None of these four genes is known to interact with each other in Arabidopsis (String-db). Despite the few genes differentially expressed in all three comparisons in both +Fe and −Fe analyses, there were a significant number of DEGs shared between HY5 vs. EV, and FIT vs. EV, comparisons in both +Fe and −Fe conditions. That silencing HY5 and FIT would affect the same genes is possible as HY5 was recently shown to positively regulate FIT in roots [31].

GO analysis allows us to determine the biological processes in plants affected by either the genotype or treatment comparisons being made. This is particularly powerful when coupled with heatmaps developed for each of the three comparisons (Figure 1 and Figure 2). Given the role of iron in photosynthesis, it is not surprising that many of the identified GO terms are associated with photosynthesis. GO terms associated with DNA replication and methylation were over-represented in both genotype and treatment analyses. The GO term with the largest number of genes associated with it was identified in cluster S6+. GO term GO:0019288, isopentenyl diphosphate biosynthesis, mevalonate-independent pathway (MEP) was associated with 102 genes. Five of the seven genes in the MEP pathway (DXS, DXR, CMK, HDS, and HDR) are easily identified in our dataset and all are down-regulated in HY5-silenced plants. Isopentenyl diphosphate is a precursor for isoprenoid compounds. Isoprenoids are very diverse and very prevalent, anywhere from 20,000 to 35,000 different isoprenoids have been identified in different species [63]. The MEP pathway is functional in the chloroplast, so the down-regulation of these genes often results in reduced chloroplast and carotenoid development [63]. The MEP pathway is also used to synthesize phytohormones including gibberellins, abscisic acid, and cytokinins [64]. The repression of genes associated with the MEP pathway may reflect the reduced photosynthesis of HY5-silenced Fiskeby III plants, even in +Fe conditions. Among the 71 over-represented GO categories identified from genes in cluster S6+, 17 are associated with photosynthesis. Two GO categories (GO:0009072 and GO0009073) are associated with aromatic amino acid (tryptophan, phenylalanine, and tyrosine) biosynthesis. Increased aromatic amino acid levels have been associated with improved abiotic stress tolerance [65,66]. Of the 35 genes associated with tyrosine, tryptophan, and phenylalanine, 32 are repressed in HY5-silenced plants under +Fe conditions compared to EV. It is possible that silencing HY5 reduces the abiotic stress tolerance through the repression of these genes. The remaining GO categories in all other clusters reflect diverse cellular processes.

## 4. Materials and Methods

### 4.1. Construct Development

Virus-induced gene silencing (VIGS) constructs were developed to knock down the expression of either GmFIT (*Glyma.13G322100*), GmHY5 (*Glyma.18G117100*), or GmPYE (*Glyma.05G027600*). These candidate genes were identified through previous experiments [5,15,16]. Reciprocal BLASTN was used to ensure these genes were the closest homolog to the Arabidopsis gene of interest. A fourth construct with no target gene insert, empty vector (EV), was used to control for viral symptoms. Silenced plants will be referred to as VIGS_FIT, VIGS_HY5, and VIGS_PYE. VIGS vectors were designed by cloning ~300 bp of the target gene of interest into the RNA2 of the bean pod mottle virus (BPMV) vector in the antisense orientation according to the protocol by Whitham et al. [67] using the following primers: GmFIT_F *GCTGTACGACTTTGTTGC*, GmFIT_R *ACTGAATGTGGAGCTTGG*, GmHY5_F *AGAGGGAGCAATAGTGG*, GmHY5_R *CGATTAATCAACCACACC*, GmPYE_F *AGATACAGGCAAGACTGG*, and GmPYE_R *TCCAAGCAGTTGTGATGG*. Due to the duplicated nature of the soybean genome, it is probable that the constructs silenced homoeologous genes within the genome. Previous studies have shown genes with >80% nucleotide identity will be silenced by a construct of interest [68]. Using BLASTN (version 2.2.29), we identified the homologs with >80% identity that are likely silenced by our constructs. Specifically, the FIT construct may also silence *Glyma.13G175200*, *Glyma.12G178500*, *Glyma.12G081200*, *Glyma.10G080900*, and *Glyma.04G034500*, while the HY5 construct might also silence *Glyma.08G302500*, and the PYE construct might also silence *Glyma.17G098900.* The two viral RNAs (RNA1 and RNA2) were used together in particle bombardment of the unifoliate Williams 82 soybean plants. VIGS infection was confirmed using a BPMV ELISA antibody assay (Agdia, Elkhart, IN, USA). Tissue from positive plants was collected, freeze-dried, and stored at −20. For this study, 150 mg of stored tissue was added to 150 mLs of PBS buffer. The tissue was disrupted in a TissueLyser II (Qiagen^®^, Germantown, MD, USA) at 80 rpm for 30 s.

### 4.2. Phenotyping VIGS Plants

For soil tests with Williams 82, seeds were germinated in Metro-Mix 900 potting soil (Sun Grow Horticulture, Agawam, MA, USA). For soil tests with Fiskeby III, seeds were germinated in grade 2 vermiculite (U-line vermiculite, Pleasant Prairie, WI, USA) for 6 days, then transferred to pots with the same soil mixture. Plants were infected with VIGS constructs when the unifoliates were fully expanded. To infect plants, carborundum was dusted over the leaves and 20 μL of the slurry was pipetted onto each of the first true leaves and manually rubbed. Plants were maintained in soil for 14 days before phenotyping. Phenotypic measurements included: SPAD readings, shoot height (from cotyledons), and shoot weight. For phenotyping in hydroponics, Fiskeby III seeds were started in vermiculite as described above. Seven days after planting (DAP), seedlings were removed from the vermiculite, triple rinsed in double distilled water, and placed into iron-sufficient (+Fe) hydroponic solutions (100 μM Fe(NO_3_)_3_) as described previously [69]. After 24 h in hydroponics, unifoliate leaves were infected with unique VIGS constructs as described above. Plants in hydroponics were maintained in +Fe solutions for 14 days after VIGS infection. On day 14, plants were rinsed in new +Fe hydroponic solution, and then half the plants were moved into new +Fe hydroponic solutions and half the plants were moved into −Fe (50 μM Fe(NO_3_)_3_) hydroponic solutions. Plants were left in their new hydroponic solutions for 21 days, at which point the phenotypic measurements described above were taken.

### 4.3. Collecting Tissue for RNAseq Analyses

For RNA-seq analyses of plants grown in hydroponics, plants were treated exactly as described for plants used for VIGS phenotyping in hydroponics, but plants were only placed in +Fe or −Fe hydroponic solutions for 60 min. After 60 min, plants were removed from hydroponics, and the third trifoliate was collected, immediately flash frozen in liquid nitrogen, and moved to −80 storage. All hydroponics plants (phenotyping and RNAseq) were maintained in the same growth chamber.

### 4.4. RNAseq Library Preparation and Transcriptomic Analyses

Total RNA was extracted using the Qiagen RNeasy Plant Mini kits (Qiagen^®^, Germantown, MD, USA). After RNA extraction, the remaining DNA was removed using the Ambion Turbo DNAse Free kit (Ambion^®^, Austin, TX, USA). The resulting RNA was further purified using the Qiagen MinElute RNA cleanup kits (Qiagen^®^, Germantown, MD, USA). Sample integrity and purity were confirmed using an Implen NanoPhotometer N60 (Implen, Munich, Germany). Samples were submitted to the Iowa State University DNA facility for sequencing. mRNA-Seq libraries were generated, and Illumina adapter sequences were added to 32 multiplex samples per lane. All samples were sequenced on the NovaSeq 6000 (Illumina^®^, San Diego, CA, USA), generating 100 bp paired-end reads. Samples were distributed across three sequencing lanes. In total, 1,095,391,734 reads were generated. The quality of these reads was confirmed using FastQC (version 0.11.9) [70]. Illumina adaptor sequences and reads <50 bp, with leading or trailing quality below 3, and when a sliding window (4 bp) average quality score <15, they were removed using Trimmomatic (version 0.39) [71]. The remaining reads were aligned to the soybean genome sequence (Gmax_508_v4.0.fa, downloaded from phytozome.net accessed on 2 March 2023) using HiSat2 (version 2.2.1) [72] and default parameters. Read counts per gene were examined to make sure the silenced gene for that sample had the highest number of reads; this is a result of viral reads with the target gene of interest and confirms VIGS infection and gene silencing. Additionally, samples were screened using FASTQ Screen (version 0.14.1) [73] to confirm viral infection. These two approaches led to the removal of a single sample EV + Fe4, as VIGS infection (and thus gene silencing ability) could not be confirmed. All remaining samples were realigned to a modified.gff file where GmFIT, GmPYE, and GmHY5 were removed (Glyma.13G322100, Glyma.05G027600, and Glyma.18G117100, respectively). This modification ensured the viral reads mapping to the target gene of interest (FIT, PYE, and HY5) were not included in the alignments because they would skew normalization and downstream statistical analyses. Each sample was aligned individually, and the output was written to SAM files. SAM files were converted to BAM files, sorted, and indexed using samtools (version 1.16) [74]. Normalized reads are available at the NCBI SRA database PRJNA1164167.

Sorted and indexed BAM files were merged and loaded into RStudio [75] for differential gene expression analysis using edgeR (version 3.18.1) [76,77]. Samples were normalized using the trimmed mean of M to account for variations in library sizes. Subsequent visualizations of biological replicates revealed two samples with altered expression patterns compared to the other three replicates. Accordingly, one VIGS_FIT and one VIGS_PYE sample (both from +Fe-grown plants) were removed. Samples were re-normalized, and differentially expressed genes were identified by comparing each target gene’s samples to the EV control samples (Tables S1, S2 and S6). An FDR of 0.05 was used to identify significantly differentially expressed genes.

Annotations were assigned to each significantly differentially expressed gene using the Gene Annotation Lookup tool at soybase.org (version 4). Significantly over-represented gene ontology (GO) terms (Corrected *p* < 0.05) associated with each of the DEG lists were identified using the GO Term Enrichment tool (version 4) at soybase.org. CLOVER [47] was used to examine the 1000 bp promoter region of each DEG for significantly over-represented (*p*-value = 0) transcription factor binding sites (TFBS) from the JASPAR 2022 plant non-redundant TFBS database [78]. Identified TFBS are provided in Table S4.

## 5. Conclusions

Based on the available literature, we believed GmFIT and GmHy5 would operate in iron-deficient conditions to induce iron stress-responses. While silencing GmFIT had no observable phenotype in Fiskeby III, our analyses demonstrate that GmFIT is a negative regulator of iron stress-responses in Fiskeby III. While silencing GmFIT did impact the expression of canonical iron uptake and homeostasis genes, it also restored the iron responsiveness of a suite of genes associated with photosynthesis and the chloroplast. Similarly, silencing the negative regulator GmHY5 resulted in the differential expression of DEGs associated with the Clark iron stress response: DNA replication/methylation, photosynthesis, stress, and defense. However, canonical iron uptake and homeostasis genes are still missing from the Fiskeby III iron stress toolbox. This suggests there must be an alternative pathway for iron uptake and utilization in Fiskeby III. We recently identified a MATE transporter that was located with an IDC QTL on chromosome 5, and was differentially expressed in response to iron stress, and when silenced in Fiskeby III resulted in chlorosis symptoms in iron-sufficient conditions [14,15,16]. Surprisingly, the. RNA-seq of VIGS plants identified only 150 DEGs. However, RNA-seq analyses across multiple timepoints and tissues confirms that Fiskeby III invokes most aspects of the Clark iron stress-responses. This suggests Fiskeby III does not induce stress-specific responses, but instead induces more general stress responses to all stresses.

## Figures and Tables

**Table 1:** The nine comparisons made in the RNAseq analyses are listed in the column Comparison. These comparisons are divided by silencing effect (VIGS vs. EV in either +Fe or −Fe) or iron stress treatment (VIGS −Fe vs. +Fe) analyses. For each comparison, the total number of differentially expressed genes (DEGs Total) identified is provided, the number of DEGs that are induced (DEGs Induced) and down-regulated (DEGs Repressed), and the number of GO terms associated with the total number of DEGs (GO Terms).

Analysis	Comparison	DEGs Total	DEGs Induced	DEGs Repressed	GO Terms
Silencing Effect	VIGS_FIT −Fe vs. EV	86	51	35	0
	VIGS_FIT +Fe vs. EV	960	637	323	52
	VIGS_HY5 −Fe vs. EV	1266	582	684	17
	VIGS_HY5 +Fe vs. EV	3553	941	2612	57
	VIGS_PYE −Fe vs. EV	21	11	10	0
	VIGS_PYE +Fe vs. EV	35	31	4	0
Trt Effect	EV: −Fe vs. +Fe	0	0	0	0
	VIGS_FIT: −Fe vs. +Fe	655	538	117	41
	VIGS_HY5: −Fe vs. +Fe	111	2	109	9
	VIGS_PYE: −Fe vs. +Fe	11	0	11	0

**Table 2:** Six of the nine comparisons (silencing effect +Fe, silencing effect −Fe, and treatment) divided into induced and repressed genes. The number of identified transcription factor binding sites (TFBS) identified by CLOVER are denoted in the TFBS column. The specific motifs can be found in Table S4. No statistically significant TFBS were identified for any of the PYE analyses due to the small number of DEGs.

Analysis	Comparison	TFBS
Silencing Effect	VIGS_HY5 vs. EV +Fe Induced	9
	VIGS_HY5 vs. EV +Fe Repressed	144
	VIGS_HY5 vs. EV −Fe Induced	58
	VIGS_HY5 vs. EV −Fe Repressed	15
Treatment Effect	VIGS_PYE −Fe vs. EV	0
	VIGS_PYE +Fe vs. EV	17
Silencing Effect	VIGS_FIT vs. EV +Fe Induced	64
	VIGS_FIT vs. EV +Fe Repressed	27
	VIGS_FIT vs. EV −Fe Induced	3
	VIGS_FIT vs. EV −Fe Repressed	3
Treatment Effect	VIGS_FIT +Fe vs. −Fe Induced	44
	VIGS_FIT +Fe vs. −Fe Repressed	2

## Data Availability

The original data presented in the study are openly available in the NCBI SRA database at https://www.ncbi.nlm.nih.gov/sra/ (accessed on 24 September 2024) using the reference PRJNA1164167.

## Supplementary Materials

The following supporting information can be downloaded at: https://www.mdpi.com/article/10.3390/ijms252111480/s1.
