# Supplementary material for: Investigating the Role of Known Arabidopsis Iron Genes in a Stress Resilient Soybean Line
Source: Int J Mol Sci. 2024 Oct 25;25(21):11480. doi: 10.3390/ijms252111480 (PMC11545859; doi:10.3390/ijms252111480)
Supplement: Supplementary file 1 [file ijms-25-11480-s001.zip › ORourke_Figure S1.pdf]

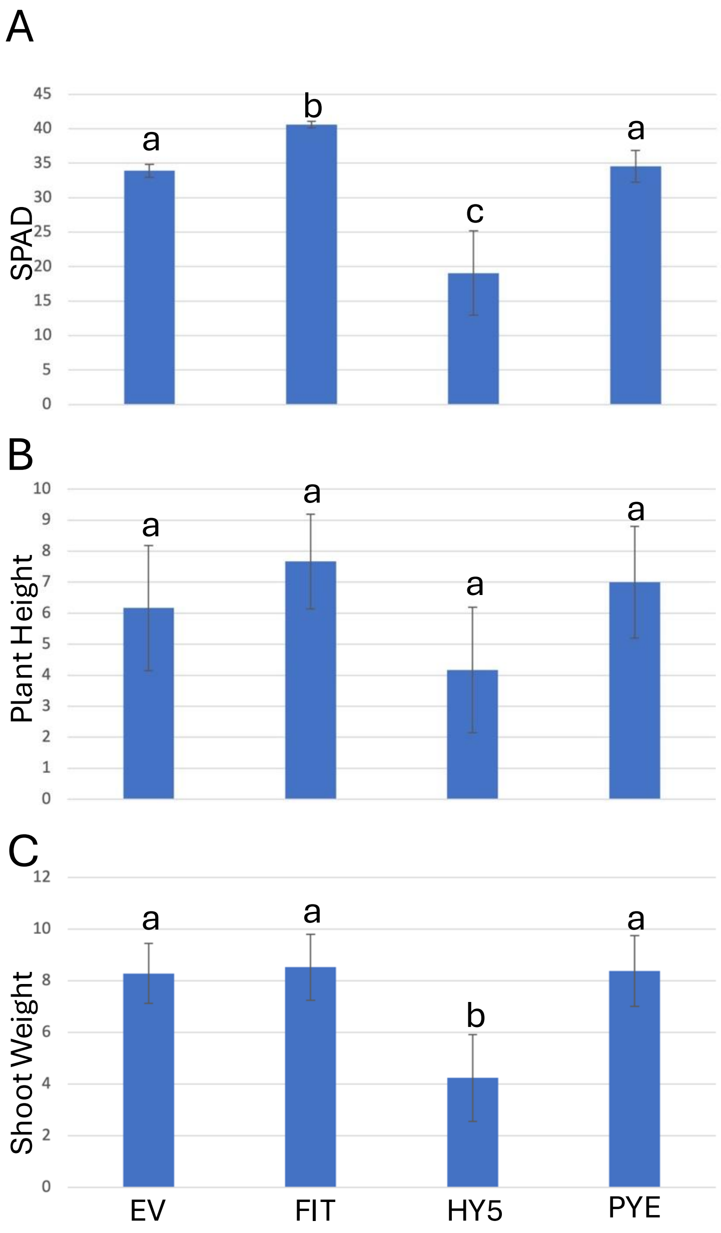


**Supplementary Figure S1.** Phenotypes of soil grown Williams 82 plants infected with either an empty vector (EV), FIT, HY5, or PYE VIGS constructs. (**A**) SPAD readings of the 2nd trifoliate 21 days post VIGS inoculation. FIT1 and HY5 are statistically significantly different from EV. (**B**) Plant height as measured from the cotyledons. None of the silenced plants differ from the EV. (**C**) Weight of stem and leaves. HY5 weighs statistically less than the EV or the other two VIGS plants.
